# Chlorido(dimethyl sulfoxide-κ*S*)[2-(2-pyrid­yl)phenyl-κ^2^ *N*,*C* ^1^]platinum(II)

**DOI:** 10.1107/S1600536808030109

**Published:** 2008-09-27

**Authors:** Masayuki Kobayashi, Shigeyuki Masaoka, Ken Sakai

**Affiliations:** a Department of Chemistry, Faculty of Science, Kyushu University, Hakozaki 6-10-1, Higashi-ku, Fukuoka 812-8581, Japan

## Abstract

In the title compound, [Pt(C_11_H_8_N)Cl(C_2_H_6_OS)], the S atom of dimethyl sulfoxide is *trans* to the pyridyl N atom [Pt—S = 2.2181 (11) Å] and the chlorido ligand is *trans* to the carbon donor of 2-(2-pyrid­yl)phenyl [Pt—Cl = 2.4202 (10) Å]. The [2-(2-pyrid­yl)phen­yl]platinum(II) unit forms a one-dimensional stack along the *c* axis with two independent inter­planar separations of 3.44 (9) and 3.50 (2) Å.

## Related literature

For background information, see: Herber *et al.* (1994 ▶); Mdleleni *et al.* (1995 ▶); Newman *et al.* (2007 ▶); Ozawa *et al.* (2006 ▶, 2007 ▶); Sakai & Ozawa (2007 ▶); Sakai *et al.* (1993 ▶); Ozawa & Sakai (2007 ▶); Kobayashi *et al.* (2008 ▶).
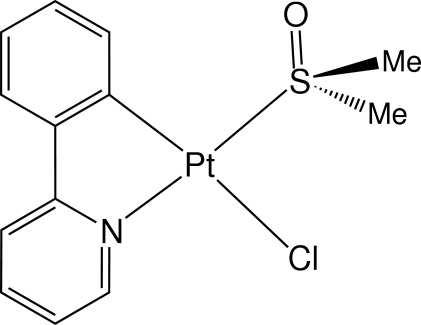


## Experimental

### 

#### Crystal data

[Pt(C_11_H_8_N)Cl(C_2_H_6_OS)]
*M* *_r_* = 462.85
Monoclinic, 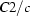

*a* = 22.414 (3) Å
*b* = 10.0205 (16) Å
*c* = 14.057 (2) Å
β = 124.512 (2)°
*V* = 2601.6 (7) Å^3^
*Z* = 8
Mo *K*α radiation
μ = 11.14 mm^−1^
*T* = 100 (2) K
0.09 × 0.08 × 0.04 mm

#### Data collection

Bruker SMART APEXII CCD-detector diffractometer
Absorption correction: multi-scan (*SADABS*; Sheldrick, 1996 ▶) *T*_min_ = 0.486, *T*_max_ = 0.640
7004 measured reflections
2850 independent reflections
2448 reflections with *I* > 2σ(*I*)
*R*_int_ = 0.018

#### Refinement

*R*[*F* ^2^ > 2σ(*F* ^2^)] = 0.023
*wR*(*F* ^2^) = 0.064
*S* = 1.11
2850 reflections
165 parameters
H-atom parameters constrained
Δρ_max_ = 2.05 e Å^−3^
Δρ_min_ = −1.43 e Å^−3^

### 

Data collection: *APEX2* (Bruker, 2006 ▶); cell refinement: *APEX2*; data reduction: *SAINT* (Bruker, 2004 ▶); program(s) used to solve structure: *SHELXS97* (Sheldrick, 2008 ▶); program(s) used to refine structure: *SHELXL97* (Sheldrick, 2008 ▶); molecular graphics: *KENX* (Sakai, 2004 ▶); software used to prepare material for publication: *SHELXL97*, *TEXSAN* (Molecular Structure Corporation, 2001 ▶), *KENX* and *ORTEPII* (Johnson, 1976 ▶).

## Supplementary Material

Crystal structure: contains datablocks global, I. DOI: 10.1107/S1600536808030109/at2633sup1.cif

Structure factors: contains datablocks I. DOI: 10.1107/S1600536808030109/at2633Isup2.hkl

Additional supplementary materials:  crystallographic information; 3D view; checkCIF report

## Figures and Tables

**Fig. 1.**
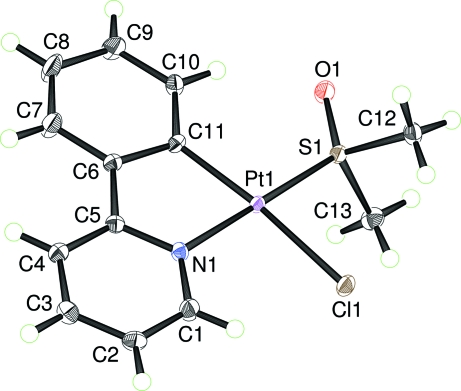
The molecular structure of (I) showing the atom-labeling scheme. Displacement ellipsoids are drawn at the 50% probability level.

**Fig. 2.**
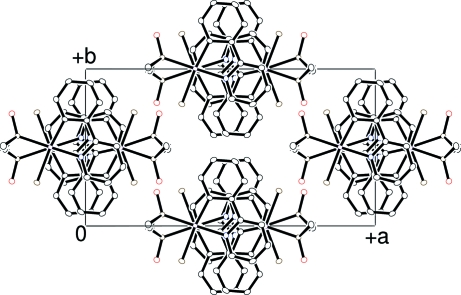
Views down the c axis, showing the manner how the phenylpyridinatoplatinum(II) units are stacked along the c axis to give a one-dimensoinal network. Hydrogen atoms are omitted for clarity.

**Fig. 3.**
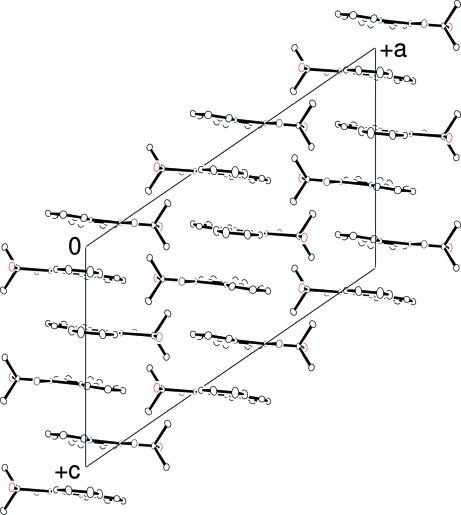
Views down the b axis, showing the manner how the phenylpyridinatoplatinum(II) units are stacked along the c axis to give a one-dimensoinal network. Hydrogen atoms are omitted for clarity.

**Fig. 4.**
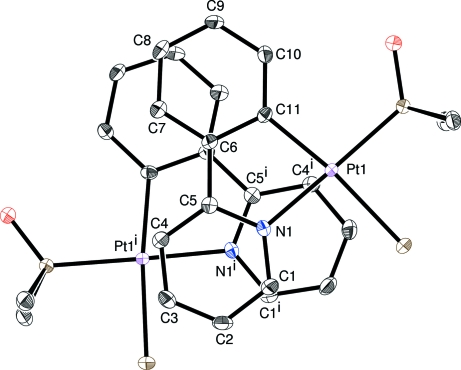
Views perpendicular to the aromatic systems that are stacked at two independent geometries [Symmetry code: (i) -x, y, 0.5 - z].

**Fig. 5.**
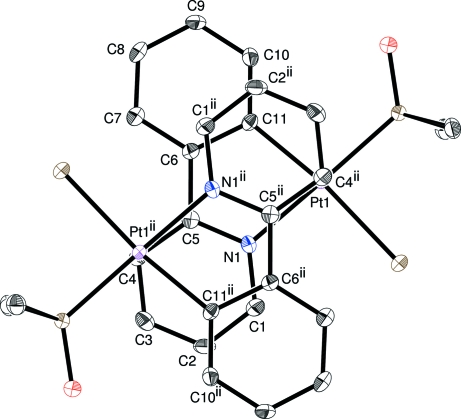
Views perpendicular to the aromatic systems that are stacked at two independent geometries [Symmetry code: (ii) -x, 1 - y, 1 - z].

## supplementary crystallographic information

### Comment

Interests over many years have concentrated on the molecular catalysis of Pt^II^ complexes in photochemical hydrogen production from water (Sakai *et al.*, 1993; Ozawa *et al.*,  2006; Sakai & Ozawa, 2007; Ozawa, Yokoyama *et al.*, 2007). The results obtained so far suggest that destabilization of the HOMO, which generally corresponds to the filled Pt^II^ d*_z_*2 orbital, gives rise to the higher H_2_-evolving activity of the complexes (Sakai & Ozawa, & Sakai, 2007). It has also been ascertained that the mononuclear Pt^II^ complexes possessing a *cis*-PtCl_2_ unit, such as *cis*-PtCl_2_(NH_3_)_2_, PtCl_2_(4,4'-dicarboxy-2,2'-bipyridine), and PtCl_2_(2,2'-bipyrimidine), exhibit considerably higher H_2_-evolving activity in comparison with those only having the amine or pyridyl type of neutral ligands, such as [Pt(NH_3_)_4_]^2+^ and [Pt(bpy)_2_]^2+^ (Ozawa, Yokoyama *et al.*, 2007). In this context, the 2-phenylpyridinate (ppy) ligand was selected because of the well known strong σ-donating character of the C(ppy) donor, expecting the higher energy level of the HOMO for the Pt^II^(ppy) complexes. As a result, the first water-soluble salt of [Pt(ppy)Cl_2_]^-^, that is, [K(18-crown-6)][Pt(ppy)Cl_2_]^.^0.5H_2_O (18-crown-6 = 1,4,7,10,13,16-hexaoxacyclooctadecane) [abbreviated as compound (II)], was recently prepared in our group and its catalytic activity in photochemical hydrogen production from water was examined in detail (Kobayashi *et al.*, in submission). The title compound, Pt(ppy)Cl(DMOS-S) (DMSO = dimethyl sulfoxide) [abbreviated as compound (I)], was first prepared from recrystallization of (II) from DMSO, but an improved synthetic route is reported in this work (see Experimental Section). It has been ascertained that the H_2_-evolving activity of (I) is much lower than that of (II), the reason for which remains ambiguous at the moment.

The donor atoms, except for the sulfur atom S1, comprise a planar geometry and the Pt atom (Pt1) does not deviate from this plane at all. The four-atom r.m.s. deviation, given in the best-plane calculation for the plane defined by atoms N1, C11, Cl1, and Pt1, was negligible (0.0003). Hereafter, this plane is defined as the Pt coordination plane. The sulfur atom (S1) and the oxygen atom (O1) of DMSO are only slightly shifted out of this plane by 0.067 (5) and 0.045 (8) Å, respectively. The torsion angles given by C11—Pt1—S1—O1 = 2.4 (2) and Cl1—Pt1—S1—O1 = -177.83 (17)° also reveal that the oxygen atom of DMSO is not largely shifted out of the coordination plane. Thus, it can be considered that (I) adopts a pseudo mirror symmetry. The benzene ring consisting of atoms C6—C11 is nearly coplanar with the coordination plane, where the dihedral angle between the benzene and the coordination planes is calculated as 0.7 (2)°. The pyridyl plane defined by atoms N1 and C1—C5 is slightly declined with respect to the coordination plane by 2.8 (2)°. The dihedral angle between the two aromatic rings is 2.5 (2)°.

The ppy ligand in compound (I, Fig. 1) does not suffer from any disorder problem. Indeed, there is a clear difference in the bond lengths of Pt—N(ppy) and Pt—C(ppy); Pt1—N1 = 2.069 (3) and Pt1—C11 = 2.002 (4) Å. Because of the strong *trans* influence originated by the C(ppy) donor, Pt1—Cl1 distance [2.4202 (10) Å] is longer than those reported for PtCl_2_(2,2'-bipyridine) [2.281 (4) - 2.306 (2) Å; Herber *et al.*, 1994]. Th Pt1—Cl1 distance is comparable to the value reported for Pt(ppy)(Hppy)Cl [2.4145 (23) Å; Mdleleni *et al.*, 1995]. In addition, the Pt1—S1 bond distance [2.2181 (11) Å], in the position *trans* to the N(ppy) donor, is comparable to those previously reported for Pt(2-(4-fluorophenyl)pyridine)Cl(DMSO) [2.2161 (16) Å; Newman *et al.*, 2007].

On the other hand, compound (I) forms a one-dimensional stack along the *c* axis based on the π-π stacking interactions between the phenylpyridinatoplatinum(II) units (see Fig. 2). The separation between the two adjacent planes is estimated as 3.44 (9) Å for the stack shown in Fig. 4 and 3.50 (2) Å for that in Fig. 5. In the former (Fig. 4), atoms C1^i^, C5^i^, N1^i^, and Pt1^i^ have an interaction to the phenylpyridinate moiety originally located and therefore shifts of these atoms from the best plane defined by atoms N1 and C1—C11 are used to calculate the separation of the two stacked planes at this geometry. In the latter (Fig. 5), atoms N1^ii^, C1^ii^, C^ii^2, C5^ii^, C6^ii^, C10^ii^, C11^ii^, and Pt1^ii^ are involved in the π-stacking association and their shifts from the best plane defined by atoms N1 and C1—C11 are similarly used to calculate the separation at this geometry. In these geometries, strong d-π interactions also contribute to the stabilization of stacking associations [Pt1—C4^i^ = 3.525 (4) and Pt1—C4^ii^ = 3.523 (4) Å; symmetry codes: (i) -*x*, *y*, 0.5 - *z*; (ii) -*x*, 1 - *y*, 1 - *z*]. Finally, it must be noted that metal-metal interactions are unimportant in this crystal [Pt1—Pt1^i^ = 5.9946 (8) and Pt1—Pt1^ii^ = 5.4225 (9) Å], where the symmetry operations are same to those given in Fig. 4 and Fig. 5.

### Experimental

A mixture of *cis*-PtCl_2_(DMSO)_2_ (0.21 g, 0.50 mmol) and 2-phenylpyridine (0.078 g, 0.50 mmol) in methanol (10 ml) was sealed in a pressure-resistant vial and was stirred at 393 K for 3 h. After the solution was cooled down to room temperature, the yellow precipitate of compound (I) was filtrated and dried *in vacuo* (Caution! Do not open the vial while it is hot, since the solution splashes out because of the violent boiling phenomenon upon a sudden decrease in pressure). Yield: 0.14 g (60%). Analysis calculated for C_13_H_14_ClNOPtS: C 33.73, H 3.05, N 3.03. Found: C 33.95, H 2.99, N 3.02. ^1^H NMR (300.53 MHz, acetone-d_6_), p.p.m.: δ 9.63 [d, J = 5.97 Hz, ^3^J(^195^Pt-^1^H) = 17.8 Hz, 1H], 8.37 [d, J = 6.81 Hz, ^3^J(^195^Pt-^1^H) = 22.8 Hz, 1H], 8.16–8.06 (m, 2H), 7.25 (d, J = 6.46 Hz), 7.48 (t, J = 6.43 Hz), 7.20–7.11 (m, 2H), 3.63 [s, ^3^J(^195^Pt-^1^H) = 12.2 Hz, 6H]. A good quality single-crystal was prepared by diffusion of methanol into a DMSO solution of (I).

### Refinement

All H atoms were placed in idealized positions (methyl C—H = 0.98 Å and aromatic C—H = 0.95 Å), and included in the refinement in a riding-model approximation, with *U*_iso_(H) = 1.5U_eq_(methyl C) and *U*_iso_(H) = 1.2U_eq_(aromatic C). In the final difference Fourier map, the highest peak was located 0.83 Å from atom Pt1. The deepest hole was located 1.07 Å from atom H9.

### Crystal data

**Table:**

[Pt(C_11_H_8_N)Cl(C_2_H_6_OS)]	*F*(000) = 1744
*M**_r_* = 462.85	*D*_x_ = 2.363 Mg m^−^^3^
Monoclinic, *C*2/*c*	Mo *K*α radiation, λ = 0.71073 Å
Hall symbol: -C 2yc	Cell parameters from 3936 reflections
*a* = 22.414 (3) Å	θ = 2.5–27.9°
*b* = 10.0205 (16) Å	µ = 11.14 mm^−^^1^
*c* = 14.057 (2) Å	*T* = 100 K
β = 124.512 (2)°	Prisms, yellow
*V* = 2601.6 (7) Å^3^	0.09 × 0.08 × 0.04 mm
*Z* = 8	

### Data collection

**Table:**

Bruker SMART APEX CCD-detector diffractometer	2850 independent reflections
Radiation source: rotating anode with a mirror focusing unit	2448 reflections with *I* > 2σ(*I*)
graphite	*R*_int_ = 0.018
φ and ω scans	θ_max_ = 27.1°, θ_min_ = 2.2°
Absorption correction: multi-scan (SADABS; Sheldrick, 1996)	*h* = −28→28
*T*_min_ = 0.486, *T*_max_ = 0.640	*k* = −12→9
7004 measured reflections	*l* = −18→15

### Refinement

**Table:**

Refinement on *F*^2^	Primary atom site location: structure-invariant direct methods
Least-squares matrix: full	Secondary atom site location: difference Fourier map
*R*[*F*^2^ > 2σ(*F*^2^)] = 0.023	Hydrogen site location: inferred from neighbouring sites
*wR*(*F*^2^) = 0.064	H-atom parameters constrained
*S* = 1.11	*w* = 1/[σ^2^(*F*_o_^2^) + (0.0295*P*)^2^ + 15.2156*P*] where *P* = (*F*_o_^2^ + 2*F*_c_^2^)/3
2850 reflections	(Δ/σ)_max_ = 0.002
165 parameters	Δρ_max_ = 2.05 e Å^−^^3^
0 restraints	Δρ_min_ = −1.43 e Å^−^^3^

### Special details

**Table:**

Experimental. The first 50 frames were rescanned at the end of data collection to evaluate any possible decay phenomenon. Since it was judged to be negligible, no decay correction was applied to the data.
Geometry. All e.s.d.'s (except the e.s.d. in the dihedral angle between two l.s. planes) are estimated using the full covariance matrix. The cell e.s.d.'s are taken into account individually in the estimation of e.s.d.'s in distances, angles and torsion angles; correlations between e.s.d.'s in cell parameters are only used when they are defined by crystal symmetry. An approximate (isotropic) treatment of cell e.s.d.'s is used for estimating e.s.d.'s involving l.s. planes.Least-squares planes (*x*,*y*,*z* in crystal coordinates) and deviations from them (* indicates atom used to define plane)-14.3368 (0.0269) *x* - 0.3580 (0.0127) *y* + 13.9887 (0.0034) *z* = 5.0906 (0.0091)* 0.0000 (0.0001) N1 * -0.0003 (0.0009) C11 * -0.0002 (0.0007) Cl1 * 0.0004 (0.0015) Pt1 - 0.0669 (0.0049) S1 - 0.0453 (0.0077) O1Rms deviation of fitted atoms = 0.0003-15.0609 (0.0266) *x* - 0.5670 (0.0181) *y* + 13.9054 (0.0039) *z* = 4.9585 (0.0082)Angle to previous plane (with approximate e.s.d.) = 2.77 (0.16)* -0.0032 (0.0026) N1 * 0.0072 (0.0030) C1 * -0.0025 (0.0030) C2 * -0.0058 (0.0029) C3 * 0.0096 (0.0029) C4 * -0.0052 (0.0027) C5 - 0.1020 (0.0055) Pt1Rms deviation of fitted atoms = 0.0061-14.3427 (0.0315) *x* - 0.4723 (0.0194) *y* + 13.9812 (0.0035) *z* = 5.0095 (0.0158)Angle to previous plane (with approximate e.s.d.) = 2.52 (0.18)* -0.0053 (0.0030) C6 * 0.0050 (0.0034) C7 * 0.0011 (0.0036) C8 * -0.0071 (0.0033) C9 * 0.0068 (0.0030) C10 * -0.0006 (0.0029) C11 0.0196 (0.0064) Pt1Rms deviation of fitted atoms = 0.0050-14.3368 (0.0269) *x* - 0.3580 (0.0127) *y* + 13.9887 (0.0034) *z* = 5.0906 (0.0091)Angle to previous plane (with approximate e.s.d.) = 0.66 (0.17)* 0.0000 (0.0001) N1 * -0.0003 (0.0009) C11 * -0.0002 (0.0007) Cl1 * 0.0004 (0.0015) Pt1 - 0.0669 (0.0049) S1 - 0.0453 (0.0077) O1Rms deviation of fitted atoms = 0.0003-14.6775 (0.0205) *x* - 0.4790 (0.0073) *y* + 13.9524 (0.0028) *z* = 4.9852 (0.0045)Angle to previous plane (with approximate e.s.d.) = 1.36 (0.14)* 0.0328 (0.0031) N1 * 0.0226 (0.0035) C1 * -0.0212 (0.0037) C2 * -0.0380 (0.0034) C3 * -0.0016 (0.0037) C4 * 0.0177 (0.0038) C5 * 0.0052 (0.0039) C6 * 0.0303 (0.0041) C7 * 0.0139 (0.0042) C8 * -0.0215 (0.0039) C9 * -0.0223 (0.0033) C10 * -0.0180 (0.0035) C11 - 3.4480 (0.0042) N1_$1 - 3.3136 (0.0062) C1_$1 - 3.5267 (0.0045) C5_$1 - 3.4606 (0.0029) Pt1_$1Rms deviation of fitted atoms = 0.0228-14.6775 (0.0205) *x* - 0.4790 (0.0073) *y* + 13.9524 (0.0028) *z* = 4.9852 (0.0045)Angle to previous plane (with approximate e.s.d.) = 0.00 (0.12)* 0.0328 (0.0031) N1 * 0.0226 (0.0035) C1 * -0.0212 (0.0037) C2 * -0.0380 (0.0034) C3 * -0.0016 (0.0037) C4 * 0.0177 (0.0038) C5 * 0.0052 (0.0039) C6 * 0.0303 (0.0041) C7 * 0.0139 (0.0042) C8 * -0.0215 (0.0039) C9 * -0.0223 (0.0033) C10 * -0.0180 (0.0035) C11 3.4700 (0.0040) N1_$2 3.4803 (0.0050) C1_$2 3.5240 (0.0050) C2_$2 3.4851 (0.0048) C5_$2 3.4977 (0.0053) C6_$2 3.5252 (0.0047) C10_$2 3.5209 (0.0046) C11_$2 3.5174 (0.0022) Pt1_$2Rms deviation of fitted atoms = 0.0228
Refinement. Refinement of *F*^2^ against ALL reflections. The weighted *R*-factor *wR* and goodness of fit *S* are based on *F*^2^, conventional *R*-factors *R* are based on *F*, with *F* set to zero for negative *F*^2^. The threshold expression of *F*^2^ > σ(*F*^2^) is used only for calculating *R*-factors(gt) *etc*. and is not relevant to the choice of reflections for refinement. *R*-factors based on *F*^2^ are statistically about twice as large as those based on *F*, and *R*- factors based on ALL data will be even larger.

### Fractional atomic coordinates and isotropic or equivalent isotropic displacement parameters (Å^2^)

**Table:**

	*x*	*y*	*z*	*U*_iso_*/*U*_eq_	
Pt1	0.121378 (8)	0.501904 (14)	0.501179 (13)	0.01100 (7)	
Cl1	0.16776 (5)	0.27601 (10)	0.54289 (9)	0.0177 (2)	
S1	0.23482 (5)	0.57296 (10)	0.61446 (9)	0.0135 (2)	
O1	0.25415 (16)	0.7153 (3)	0.6395 (3)	0.0207 (7)	
N1	0.01517 (18)	0.4393 (4)	0.3907 (3)	0.0130 (7)	
C1	−0.0056 (2)	0.3097 (4)	0.3637 (4)	0.0188 (9)	
H1	0.0298	0.2412	0.4003	0.023*	
C2	−0.0769 (2)	0.2759 (4)	0.2843 (4)	0.0198 (9)	
H2	−0.0905	0.1849	0.2656	0.024*	
C3	−0.1290 (2)	0.3759 (5)	0.2317 (4)	0.0179 (9)	
H3	−0.1784	0.3542	0.1764	0.021*	
C4	−0.1079 (3)	0.5076 (4)	0.2611 (4)	0.0156 (9)	
H4	−0.1428	0.5771	0.2276	0.019*	
C5	−0.0353 (2)	0.5373 (4)	0.3399 (4)	0.0128 (8)	
C6	−0.0046 (2)	0.6714 (4)	0.3759 (4)	0.0141 (8)	
C7	−0.0479 (2)	0.7861 (4)	0.3361 (4)	0.0226 (10)	
H7	−0.0990	0.7784	0.2841	0.027*	
C8	−0.0161 (3)	0.9104 (5)	0.3726 (5)	0.0286 (11)	
H8	−0.0453	0.9885	0.3455	0.034*	
C9	0.0584 (2)	0.9211 (4)	0.4488 (4)	0.0216 (9)	
H9	0.0804	1.0066	0.4732	0.026*	
C10	0.1010 (2)	0.8066 (4)	0.4897 (4)	0.0158 (8)	
H10	0.1519	0.8155	0.5433	0.019*	
C11	0.0714 (2)	0.6793 (4)	0.4545 (4)	0.0128 (8)	
C12	0.2791 (2)	0.4911 (4)	0.7508 (4)	0.0183 (9)	
H12A	0.3312	0.5089	0.7948	0.027*	
H12B	0.2706	0.3948	0.7389	0.027*	
H12C	0.2598	0.5247	0.7938	0.027*	
C13	0.2836 (3)	0.5101 (4)	0.5582 (4)	0.0198 (10)	
H13A	0.2636	0.5486	0.4816	0.030*	
H13B	0.2791	0.4127	0.5519	0.030*	
H13C	0.3348	0.5346	0.6101	0.030*	

### Atomic displacement parameters (Å^2^)

**Table:**

	*U*^11^	*U*^22^	*U*^33^	*U*^12^	*U*^13^	*U*^23^
Pt1	0.01044 (10)	0.00879 (10)	0.01265 (11)	0.00073 (6)	0.00587 (8)	0.00039 (5)
Cl1	0.0155 (5)	0.0109 (4)	0.0218 (5)	0.0024 (4)	0.0076 (4)	0.0010 (4)
S1	0.0114 (5)	0.0116 (5)	0.0152 (5)	0.0004 (4)	0.0062 (4)	−0.0005 (4)
O1	0.0144 (15)	0.0135 (15)	0.0254 (17)	−0.0006 (12)	0.0061 (13)	−0.0018 (13)
N1	0.0105 (16)	0.0156 (17)	0.0126 (16)	0.0001 (15)	0.0064 (14)	0.0002 (14)
C1	0.019 (2)	0.013 (2)	0.020 (2)	0.0007 (17)	0.0082 (19)	0.0022 (17)
C2	0.022 (2)	0.013 (2)	0.023 (2)	−0.0050 (18)	0.012 (2)	−0.0042 (17)
C3	0.014 (2)	0.021 (2)	0.019 (2)	−0.0037 (17)	0.0096 (18)	−0.0006 (17)
C4	0.017 (2)	0.014 (2)	0.017 (2)	0.0010 (16)	0.0104 (19)	0.0008 (15)
C5	0.015 (2)	0.0156 (19)	0.0109 (19)	0.0004 (17)	0.0088 (17)	0.0003 (16)
C6	0.015 (2)	0.012 (2)	0.015 (2)	0.0003 (16)	0.0081 (17)	−0.0006 (15)
C7	0.017 (2)	0.015 (2)	0.032 (3)	0.0022 (18)	0.012 (2)	0.0014 (19)
C8	0.021 (2)	0.014 (2)	0.041 (3)	0.0067 (18)	0.012 (2)	0.003 (2)
C9	0.018 (2)	0.012 (2)	0.033 (3)	−0.0042 (18)	0.013 (2)	−0.0027 (19)
C10	0.0127 (19)	0.017 (2)	0.016 (2)	0.0009 (17)	0.0064 (17)	0.0016 (16)
C11	0.014 (2)	0.0118 (18)	0.015 (2)	0.0023 (16)	0.0100 (17)	0.0014 (16)
C12	0.013 (2)	0.021 (2)	0.017 (2)	0.0016 (16)	0.0062 (19)	0.0009 (16)
C13	0.017 (2)	0.021 (2)	0.025 (2)	−0.0001 (17)	0.014 (2)	−0.0019 (17)

### Geometric parameters (Å, °)

**Table:**

Pt1—C11	2.002 (4)	C3—H3	0.9500
Pt1—N1	2.069 (3)	C4—H4	0.9500
Pt1—S1	2.2181 (11)	C5—C6	1.464 (6)
Pt1—Cl1	2.4202 (10)	C6—C7	1.400 (6)
S1—O1	1.474 (3)	C6—C11	1.413 (6)
S1—C12	1.782 (5)	C7—C8	1.382 (6)
S1—C13	1.788 (5)	C7—H7	0.9500
N1—C5	1.355 (6)	C8—C9	1.386 (6)
N1—C1	1.359 (6)	C8—H8	0.9500
C1—C2	1.377 (6)	C9—C10	1.392 (6)
C2—C3	1.392 (6)	C9—H9	0.9500
C3—C4	1.384 (6)	C10—C11	1.393 (6)
C4—C5	1.385 (6)	C10—H10	0.9500
Pt1—C4^i^	3.525 (4)	C12—H12A	0.9800
Pt1—C4^ii^	3.523 (4)	C12—H12B	0.9800
Pt1—Pt1^i^	5.9946 (8)	C12—H12C	0.9800
Pt1—Pt1^ii^	5.4225 (9)	C13—H13A	0.9800
C1—H1	0.9500	C13—H13B	0.9800
C2—H2	0.9500	C13—H13C	0.9800
			
C11—Pt1—N1	80.28 (16)	C7—C6—C11	121.5 (4)
C11—Pt1—S1	98.69 (12)	C7—C6—C5	122.1 (4)
N1—Pt1—S1	177.97 (10)	C11—C6—C5	116.3 (4)
C11—Pt1—Cl1	173.26 (12)	C8—C7—C6	119.8 (4)
N1—Pt1—Cl1	92.98 (10)	C8—C7—H7	120.1
S1—Pt1—Cl1	88.05 (4)	C6—C7—H7	120.1
O1—S1—C12	106.22 (19)	C7—C8—C9	119.9 (4)
O1—S1—C13	106.0 (2)	C7—C8—H8	120.0
C12—S1—C13	101.9 (2)	C9—C8—H8	120.0
O1—S1—Pt1	122.98 (13)	C8—C9—C10	120.0 (4)
C12—S1—Pt1	109.74 (15)	C8—C9—H9	120.0
C13—S1—Pt1	107.97 (16)	C10—C9—H9	120.0
C5—N1—C1	119.6 (4)	C9—C10—C11	122.1 (4)
C5—N1—Pt1	116.0 (3)	C9—C10—H10	119.0
C1—N1—Pt1	124.4 (3)	C11—C10—H10	119.0
N1—C1—C2	121.2 (4)	C10—C11—C6	116.7 (4)
N1—C1—H1	119.4	C10—C11—Pt1	129.1 (3)
C2—C1—H1	119.4	C6—C11—Pt1	114.2 (3)
C1—C2—C3	119.5 (4)	S1—C12—H12A	109.5
C1—C2—H2	120.2	S1—C12—H12B	109.5
C3—C2—H2	120.2	H12A—C12—H12B	109.5
C4—C3—C2	119.1 (4)	S1—C12—H12C	109.5
C4—C3—H3	120.5	H12A—C12—H12C	109.5
C2—C3—H3	120.5	H12B—C12—H12C	109.5
C3—C4—C5	119.5 (4)	S1—C13—H13A	109.5
C3—C4—H4	120.3	S1—C13—H13B	109.5
C5—C4—H4	120.3	H13A—C13—H13B	109.5
N1—C5—C4	121.2 (4)	S1—C13—H13C	109.5
N1—C5—C6	113.2 (4)	H13A—C13—H13C	109.5
C4—C5—C6	125.6 (4)	H13B—C13—H13C	109.5
			
C11—Pt1—S1—O1	2.4 (2)	C4—C5—C6—C7	2.9 (7)
Cl1—Pt1—S1—O1	−177.83 (17)	N1—C5—C6—C11	1.3 (5)
C5—N1—C1—C2	0.9 (6)	C4—C5—C6—C11	−177.9 (4)
N1—C1—C2—C3	−0.8 (7)	C11—C6—C7—C8	0.9 (7)
C1—C2—C3—C4	−0.4 (6)	C5—C6—C7—C8	−179.9 (4)
C2—C3—C4—C5	1.6 (6)	C6—C7—C8—C9	−0.3 (8)
C1—N1—C5—C4	0.3 (6)	C7—C8—C9—C10	−0.9 (7)
C1—N1—C5—C6	−178.9 (4)	C8—C9—C10—C11	1.4 (7)
C3—C4—C5—N1	−1.5 (6)	C9—C10—C11—C6	−0.8 (6)
C3—C4—C5—C6	177.5 (4)	C7—C6—C11—C10	−0.4 (6)
N1—C5—C6—C7	−178.0 (4)	C5—C6—C11—C10	−179.6 (4)

Symmetry codes: (i) −*x*, *y*, −*z*+1/2; (ii) −*x*, −*y*+1, −*z*+1.
